# Immobilizing hydroxycholesterol with apatite on titanium surfaces to induce ossification

**DOI:** 10.1186/2055-7124-18-16

**Published:** 2014-10-20

**Authors:** Cen Chen, Hyeong Cheol Yang, In-Seop Lee

**Affiliations:** Bio-X Center, College of Life Sciences, Zhejiang Sci-Tech University, Hangzhou, 310018 China; Department of Dental Biomaterials Science, Seoul National University, Seoul, 110-749 Korea; Institute of Natural Sciences, Yonsei University, Seoul, 120-749 Korea

**Keywords:** Hydroxycholesterol, Apatite, Titanium, Ossification

## Abstract

**Background:**

Immobilizing bioactive molecules and osteoconductive apatite on titanium implants have investigated direct ossification. In this study, hydroxycholesterol (HC) was immobilized with apatite on titanium through simply adsorption or sandwich-like coating. Three kinds of hydroxycholesterol were chosen to induce ossification: 20α-hydroxycholesterol (20α- HC), 22(S)-hydroxycholesterol (22(S)-HC) and 25-hydroxycholesterol (25-HC).The effects of HC/apatite coating on ossification abilities were evaluated *in vitro* and *in vivo*.

**Results:**

At 6 d, adsorbed apatite/25-HC and apatite/22(S)-HC coating exhibited some cytotoxicity, while the cell viability of apatite/20α-HC coating was similar as apatite coating. Immobilizing HC with apatite significantly enhanced the ALP activities compared with apatite coating. There was no significant difference in ALP value between adsorbed apatite/HC coating and sandwich-like apatite/HC/apatite coating. When compared with apatite coating, the mineral deposition improved by adsorbed HC with apatite at higher concentration *in vivo*.

**Conclusions:**

When compared with apatite coating, immobilizing HC with apatite coating induced the ossification *in vitro* and *in vivo*.

## Background

Titanium (Ti) implants are widely used in orthopaedic surgery and dentistry because of favourable mechanical and biocompatible properties [1, 2]. Numerous studies have reported that the early events of bone healing around implants are strongly related to their long-term clinical success [3]. In order to induce the direct ossification of implants, various surface treatments have been developed [4–6]. These treatments include modifications in their surface properties or coating various calcium phosphates (CaP) including a biomimetic apatite layer. The biomimetic precipitation whereby such apatite layers are produced, has profound consequences for their potential to serve carriers for bioactive molecules [7, 8], and control the release of loaded molecules [9].

Over the last decades, a variety of bioactive molecules have been immobilized with apatite layer to facilitate ossification [10–12]. Among all these researches, bone morphogenic proteins (BMPs) have drawn lots of attraction [13, 14]. While BMPs have exhibited clinical efficacy in early osseointegration, its potential for widespread application is limited by its high cost and its side effects [15].

Hydroxycholesterols (HC), also known oxysterols are oxidized derivatives of cholesterol found naturally in tissues and circulatory systems of mammal [16]. The HCs are involved in different biological procedures, including cholesterol homeostasis, sphingolipid metabolism, platelet aggregation, and apoptosis [17]. In particular, the naturally occurring 25-, 22(S)-, and 20α- hydroxycholesterol analogues have been demonstrated to induce osteogenic differentiation in primary murine mesenchymal stem cells [17], and also showed successful bone regeneration in a mouse spinal fusion when HC was delivered to the defect site [18].

There are many methods for loading bioactive molecules with biomimetically formed apatite layer such as physical adsorption, covalent binding, and biomimetic coprecipitation [19]. As a derivative of cholesterol, HCs are hydrophobic, and as such when immobilized with apatite coating via coprecipitation, they are not efficient to induce osteogenic differentiation of fibroblast cells (C3H10T1/2) [20].

In the present study, we deposited a calcium phosphate layer on titanium discs by ion-beam assisted deposition (IBAD), and use such deposited layer as active substrates to biomimetically prepare apatite coating in Dulbecco’s phosphate buffered saline solution containing CaCl_2_. Then, HC was immobilized with apatite on Ti substrates through simply adsorption or a sandwich-like coating, the ossification of the apatite coated Ti with or without HC was investigated *in vitro* and *in vivo*.

## Methods

### Preparation of CaP deposited Ti substrate

Commercially pure titanium (grade IV) were obtained from Supra Alloys Inc. (Camarillo, CA, USA), and cut into discs (10 mm in diameter and 2 mm in thickness) in Dentium Co., Ltd. The surfaces of Ti discs were machined, and washed in acetone and distilled water ultrasonically to be used as substrates. Thin calcium phosphate films with a thickness of 500 nm were deposited by ion-beam assisted deposition (CaP-Ti). The details of CaP deposition through IBAD have been described elsewhere [21]. Briefly, evaporants of CaP were prepared by sintering the powder mixtures of hydroxyapatite (Alfa, USA) and CaO (Cerac, USA) at 1200°C in air for 2 h. For CaP deposition, an electron beam evaporator (Telemark, USA) and an end-hall type ion gun (Commonwealth Scientific, USA) were employed. Heat treatments after the deposition were conducted at 350°C with the heating rate of 5°C/min and held for 1 h, and then cooled to room temperature in furnace.

### Solutions and hydroxycholesterol used

Reagent grade CaCl_2_ (100 mg/L) was dissolved in Dulbecco’s phosphate buffered saline (calcium/magnesium free) to prepare the DPBS solution. Hydroxycholesterol was purchased from Sigma-Aldrich (St Louis, MO). Three kinds of hydroxycholesterol were chosen for direct ossification: 20α- hydroxycholesterol (20α-HC), 22(S)-hydroxycholesterol (22(S)-HC) and 25-hydroxycholesterol (25-HC). Hydroxycholesterol was dissolved in 100% ethanol (EtOH) (1 mg/mL) to prepare the working reagent. The DPBS was sterilized by filtration using a membrane with a pore size of 0.20 μm before use.

### Immobilizing hydroxycholesterol with apatite on CaP deposited Ti discs

**Figure 1:**
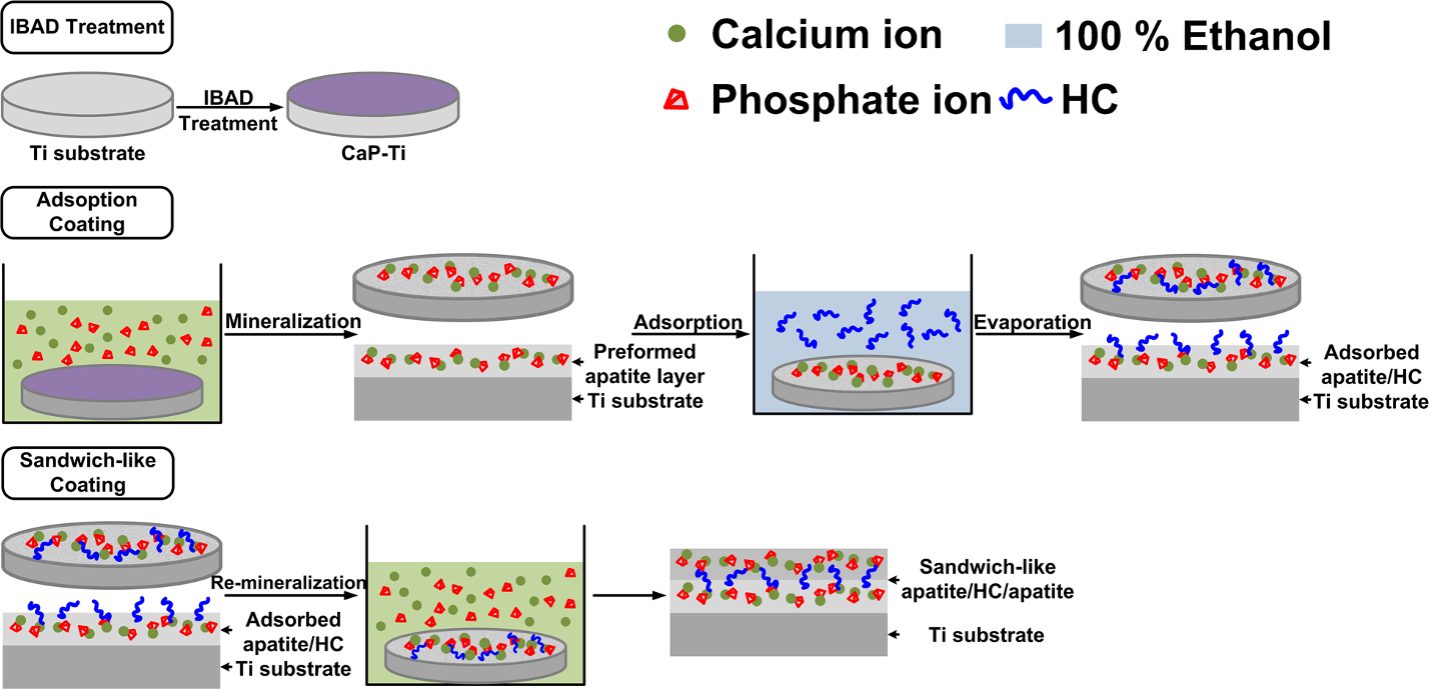
**Sketch map of immobilizing hydroxycholesterol with apatite on titanium surfaces.**

Figure 1 shows two methods to immobilize HC with apatite on CaP-Ti, adsorption, and sandwich-like coating. Before immobilizing the HC, each CaP-Ti was sterilized in 70% ethanol, and distilled water, then place under UV light over night. For adsorbed apatite/HC coating, sterilized CaP-Ti was firstly immersed in DPBS at 37°C for 24 h to prepare the preformed apatite layer. Then the apatite coated Ti was dipped in 1 ml of 100% EtOH containing 1 mg HC for 10 s and then dried in air. For sandwich-like apatite/HC/apatite coating, the Ti with adsorbed apatite/HC coating was again immersed in DPBS for another 24 h to form the second apatite layer. As the water solubility of HC is very low, we speculated that the HC adsorbed on the preformed apatite layer could almost retain during the second apatite formation.

### Cell culture

Mouse embryo fibroblast cells (C3H10T1/2) were obtained from the American Type Culture Collection (ATCC, Rockville, MD) and cultured in growth medium (Gibco-BRL) under a standard cell culture conditions (a sterile, 37°C, humidified, 5% CO2 environment). The growth medium was composed of Basal Medium Eagle (BME), 4.5 g/L of glucose, 10% fetal bovine serum (FBS) and antibiotic solutions (100 U/mL of penicillin-G and 100 μg/mL of streptomycin). The growth medium was changed every 3 d until the cells reached 80–100% confluence. For evaluation the effects of hydroxycholesterol *in vitro*, C3H10T1/2 cells were seeded on samples placed into 48-well culture plates (n =3 per group). For control experiments, cells were seeded on apatite coated CaP-Ti without HC.

### Cells viability

C3H10T1/2 cells viability was quantitatively evaluated by measuring the total dsDNA amount described earlier [22]. C3H10T1/2 cells were seeded on samples in growth medium for 6 d. At determined time, cells were washed twice with PBS, and lysed utilizing a buffer containing 0.5% Triton X-100. The total dsDNA in the lysate was measured using the quant-iT™ PicoGreen dsDNA reagent and kits (Molecular Probes, USA) according to the protocol from the manufacturer. The fluorescence at wavelengths of 480/520 nm was determined using a fluorescence microplate reader (Fluostar OPTIMA, Germany) and DNA quantity was determined by using standard DNA dilution series.

### Alkaline phosphatase activity assay

The Alkaline phosphatase (ALP) activity of C3H10T1/2 cells on each sample was measured using a 4-nitrophenyl phosphate colorimetric assay [23]. The cells were seeded on samples in growth medium or mineralizing medium (BME containing FBS, glucose, penicillin-streptomycin, ascorbate, and β-glycerophosphate), and the medium was changed every 3 d. After 6 d of cultivation, the cells on each sample were washed gently, and incubated in the mixture solution of 140 μL alkaline buffer, 10 μL of 1.5 M MgCl_2_ and 67 mM 4-nitrophenyl phosphate (Fluka, Buchs, Switzerland) at 37°C for 30 min. The reaction was stopped using 0.2 M NaOH. The absorbance was measured at 405 nm using a microplate reader. ALP activity was calculated from a standard curve after normalizing to the total protein content, which was measured using Micro-BCA protein assay kit (Pierce, USA). ALP activity was expressed as units per mg protein.

### Mineralization on Ti in a rat model

**Figure 2:**
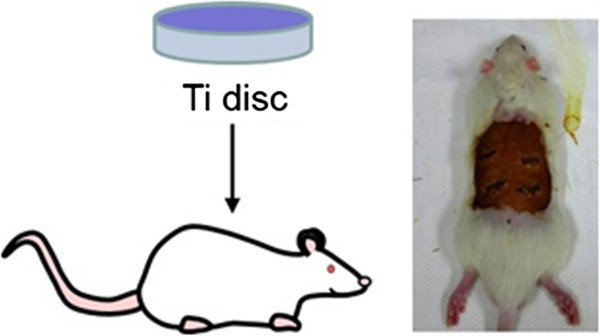
**Sketch map of Ti discs implantation into rat muscle beds.**

The effect of hydroxycholesterol on mineralization deposition *in vivo* was investigated in a rat model. Twelve six-week old SD male rats, weighing 200–250 g, were used in this study. Animal experiments were carried out with the approval of the animal welfare committee of Seoul National University Institutional Animal Care and Use Committe (Approval No: SNU-091231-1). Following general anesthesia, four subcutaneous pockets at dorsal skin of each rat were made by blunt dissection as shown in Figure 2. Apatite coated Ti discs with or without HC were implanted into muscle beds of subcutaneous pockets. After implantation for 5 weeks, the rats were sacrificed humanely, and then Ti discs were removed and stained with Alizarin Red S.

### Statistical analysis

All quantitative data were depicted as the mean ± standard deviation (n =3). Tests of significance were performed using Student’s t-test.

## Results and discussion

### Effects of hydroxycholesterol on osteogenic differentiation *in vitro*

To investigate the effects of hydroxycholesterol on osteogenic differentiation of C3H10T1/2, the cells were grown on Ti substrates with adsorbed apatite/25-HC, apatite/22(S)-HC, apatite/20α-HC coatings in growth medium for 6 days, and then the cell cytotoxicity together with ALP activity were evaluated.

**Figure 3:**
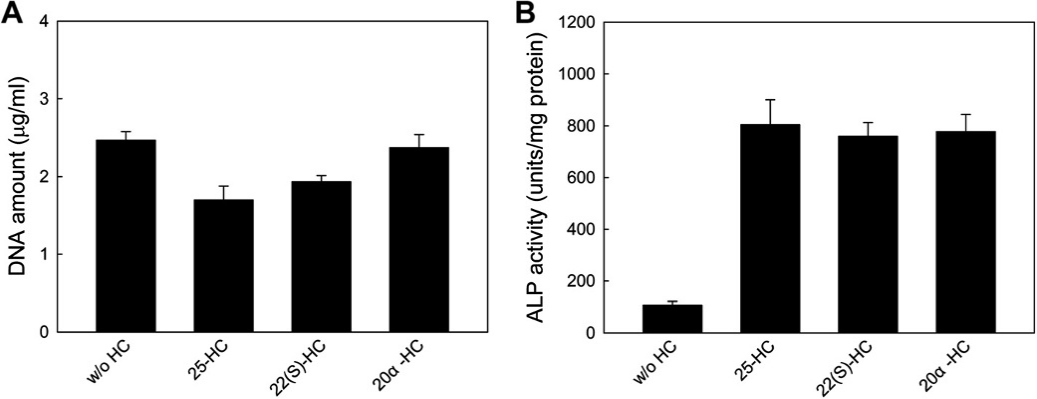
**Effects of hydroxycholesterol on (A) cell viability and (B) ALP activity.** C3H10T1/2 cells were cultured on samples with apatite coating or adsorbed apatite/HC coating in growth medium for 6 d. Three kinds of hydroxycholesterol were chosen to prepare the adsorbed apatite/HC coating: 25-HC, 22(S)-HC, and 20α-HC. The values represent the mean ± standard deviation (n =3).

From the total DNA amount data (Figure 3a), apatite/25-HC coating exhibited the most cytotoxicity, followed by apatite/22(S)-HC and apatite/20α-HC coatings. From the literature [24], the apoptosis effect of 25-HC could be inhibited by calcium ion channel blockers, which indicates that the calcium ion influx through ion channels on plasma membrane leads to the depressing of oxysterol-induced apoptosis. Since the coating layer on Ti discs contained apatite, which was the rich source of calcium ions, the cytotoxicity of HC coating was not as high as previous reports [25].

As shown in Figure 3b, the ALP activities on apatite/HC coating layers significantly improved compared to apatite coating without HC (*p* <0.05), and there was no significant difference among apatite/25-HC, apatite/22(S)-HC and apatite/20α-HC coatings (*p* >0.05). As the derivative of cholesterol, hydroxycholesterols are hydrophobic, which are not easy to coprecipitate with apatite on Ti substrate [20] and also encounter with formulation-related issues for clinical application [16]. In our study, although the HC was simply adsorbed on Ti substrates with apatite, the apatite/HC coating layer demonstrated higher ALP activity of C3H10T1/2 cells. This result shows that immobilizing HC onto apatite dos not interfere with its molecular properties. Based on the results of cytotoxicity and osteogenic differentiation ability, 20α-HC was chosen for the following investigation.

### Effects of methods to immobilize hydroxycholesterol on ALP activities *in vitro*

**Figure 4:**
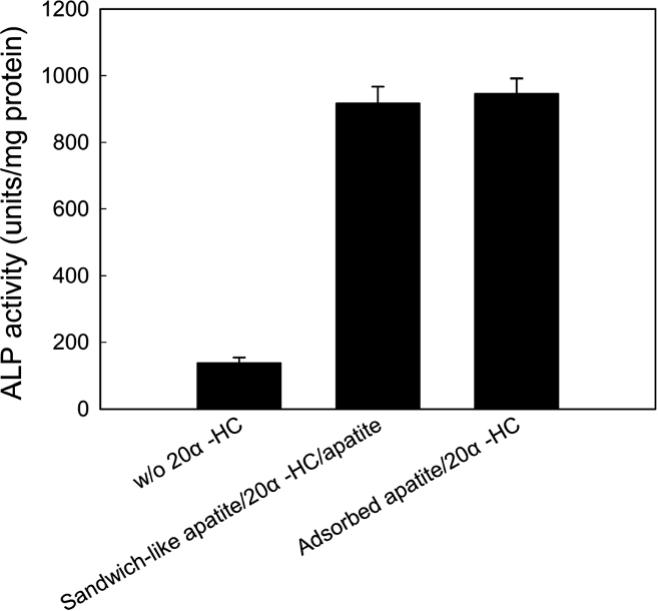
**Normalized ALP activities with respect to total protein at day 6.** C3H10T1/2 cells were seeded on samples with apatite coating, sandwich-like apatite/20α-HC/apatite coating, and adsorbed apatite/20α-HC coating in mineralizing medium for 6 d. The values represent the mean ± standard deviation (n =3).

The naturally occurring hydroxycholesterol analogues have shown to induce osteogenic differentiation *in vitro and in vivo* [26]. However, they are cytotoxic at high concentration [27], which indicates that the working doses need to be controlled. The biomimetic apatite coatings can serve as carriers to control release immobilized HC. In this study, we modified Ti substrate with adsorbed apatite/20α-HC or sandwich-like apatite/20α-HC/apatite coatings, and investigated the effects of these two methods on ALP activities of C3H10T1/2. Cells were seeded on samples in mineralizing medium, and the ALP activities of C3H10T1/2 cells were determined at day 6 (Figure 4).

The ALP activities were extremely enhanced by introducing 20α-HC to apatite coating (*p* <0.05), while no significant difference between adsorbed apatite/20α-HC coating and sandwich-like apatite/20α-HC/apatite coating (*p* >0.05). From the results of ALP activity, sandwich-like apatite/20α-HC/apatite demonstrated bioactivity similar to adsorbed apatite/20α-HC, which suggests that 20α-HC remained on the preformed first apatite layer during washing and re-mineralization of the second apatite layer. Interestingly, the second apatite layer could trap 20α-HC into two apatite layers for a more sustained release [28], but the ALP level of C3H10T1/2 was not affected much. ALP activities were enhanced by HC in a dose-dependent manner [20], while the effects of dose was less significant at higher concentration. At day 6, the released 20α-HC from both coating layers in culture medium might be higher than the threshold concentration, which made it difficult to relate release concentration to ALP activities.

### Effects of hydroxycholesterol on mineral deposition *in vivo*

**Figure 5:**
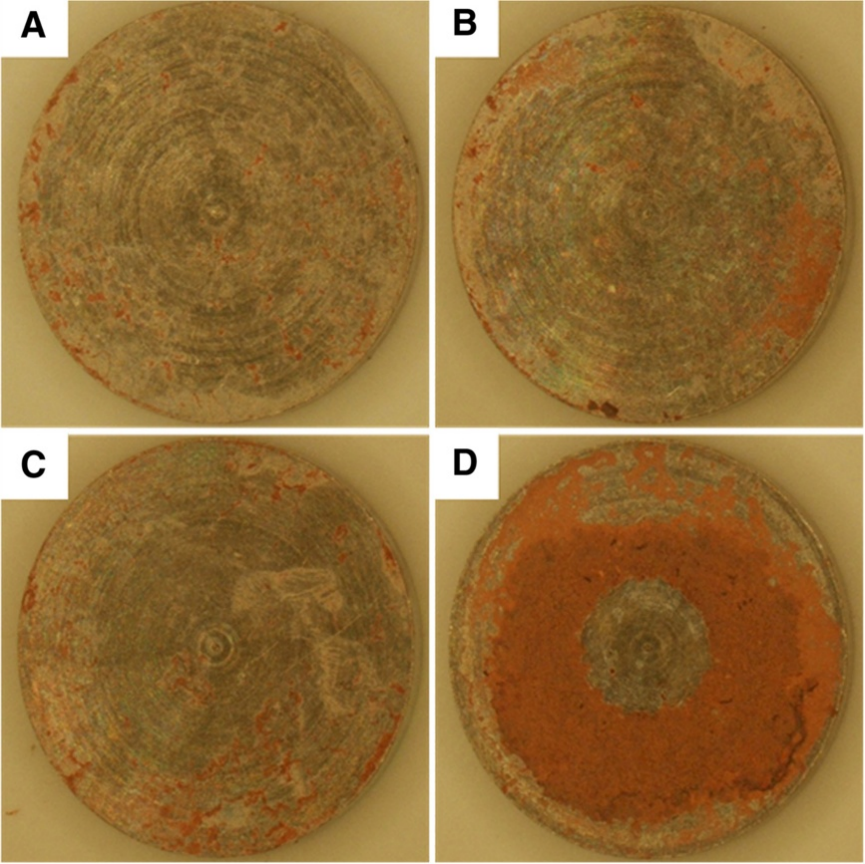
**Effects of adsorbed apatite/20α-HC coating on mineral deposition in rat model.** Experimental groups were set up: **(A)** CaP-Ti discs were immersed in DPBS solution for 24 h to prepare apatite coating. Apatite coated CaP-Ti discs were immersed in 100% ethanol containing **(B)** 1 mg/mL, **(C)** 2 mg/mL and **(D)** 4 mg/mL 20α-HC for 10 s and then dried in air to prepare adsorbed apatite/20α-HC coating. All the experiment groups were subcutaneously implanted into rat muscle bed for 5 weeks, and then the deposited mineral on surfaces of samples were stained with Alizarin Red S.

Given the greater ALP activity of immobilizing apatite coating with HC as compared with apatite coating, we anticipated that immobilizing apatite with HC on CaP-Ti would enhance mineral deposition on implanted titanium substrates. To test this hypothesis, we implanted apatite coated CaP-Ti with or without adsorbed 20α-HC into a rat model for 5 weeks. The samples with adsorbed apatite/20α-HC coating were prepared as previously stated in above section using 1, 2, and 4 mg/mL of 20α-HC in 100% EtOH. Through the Alizarin Red S staining, we observed the mineral deposition *in vivo*. The mineralization was limited to the boundary of apatite coated Ti discs (Figure 5a). With immobilizing 20α-HC at lower concentrations, no significant difference could be observed compared with only apatite coated CaP-Ti (Figure 5b, and c). However, at the higher concentration of 4 mg/mL of 20α-HC, mineral deposition was greatly enhanced and occurred more evenly over titanium substrates (Figure 5d). These results strongly support the immobilizing apatite with HC on Ti surfaces to induce direct ossification of Ti implants.

## Conclusions

Adsorbed 25-HC with apatite presented higher cytotoxicity, followed by 22(S)-HC and 20α-HC. Immobilizing HC with apatite by simply adsorption and sandwich-like coating increased the ALP activity of C3H10T1/2, but there was no difference between apatite/HC and apatite/HC/apatite coatings. With immobilizing 20α-HC at the concentration of 4 mg/mL led to a higher and more uniform mineral deposition in rat model. Immobilizing hydroxycholesterol with apatite on titanium implants would have some positive effects on direct ossification.

## References

[1] Ribeiro AL, Hammer P, Vaz LG, Rocha LA (2013). Are new TiNbZr alloys potential substitutes of the Ti6Al4V alloy for dental applications? An electrochemical corrosion study. Biomed Mater.

[2] Andersen OZ, Offermanns V, Sillassen M, Almtoft KP, Andersen IH, Sorensen S, Jeppesen CS, Kraft DC, Bottiger J, Rasse M, Kloss F, Foss M (2013). Accelerated bone ingrowth by local delivery of strontium from surface functionalized titanium implants. Biomaterials.

[3] Lavenus S, Berreur M, Trichet V, Pilet P, Louarn G, Layrolle P (2011). Adhesion and Osteogenic Differentiation of Human Mesenchymal Stem Cells on Titanium Nanopores. Eur Cell Mater.

[4] Violant D, Galofre M, Nart J, Teles RP (2014). In vitro evaluation of a multispecies oral biofilm on different implant surfaces. Biomed Mater.

[5] Nayak S, Dey T, Naskar D, Kundu SC (2013). The promotion of osseointegration of titanium surfaces by coating with silk protein sericin. Biomaterials.

[6] Bayram C, Demirbilek M, Yalcin E, Bozkurt M, Dogan M, Denkbas EB (2014). Osteoblast response on co-modified titanium surfaces via anodization and electrospinning. Appl Surf Sci.

[7] Yazaki Y, Oyane A, Sogo Y, Ito A, Yamazaki A, Tsurushima H (2011). Control of gene transfer on a DNA-fibronectin-apatite composite layer by the incorporation of carbonate and fluoride ions. Biomaterials.

[8] Wang XP, Oyane A, Tsurushima H, Sogo Y, Li X, Ito A (2011). BMP-2 and ALP gene expression induced by a BMP-2 gene-fibronectin-apatite composite layer. Biomed Mater.

[9] Bae SE, Choi J, Joung YK, Park K, Han DK (2012). Controlled release of bone morphogenetic protein (BMP)-2 from nanocomplex incorporated on hydroxyapatite-formed titanium surface. J Control Release.

[10] Lee HJ, Koo AN, Lee SW, Lee MH, Lee SC (2013). Catechol-functionalized adhesive polymer nanoparticles for controlled local release of bone morphogenetic protein-2 from titanium surface. J Control Release.

[11] Yan SG, Zhang J, Tu QS, Ye JH, Luo E, Schuler M, Kim MS, Griffin T, Zhao J, Duan XJ, Cochran DJ, Murray D, Yang PS, Chen J (2011). Enhanced osseointegration of titanium implant through the local delivery of transcription factor SATB2. Biomaterials.

[12] Chen C, Lee IS, Zhang SM, Yang HC (2010). Biomimetic apatite formation on calcium phosphate-coated titanium in Dulbecco's phosphate-buffered saline solution containing CaCl(2) with and without fibronectin. Acta Biomater.

[13] Guillot R, Gilde F, Becquart P, Sailhan F, Lapeyrere A, Logeart-Avramoglou D, Picart C (2013). The stability of BMP loaded polyelectrolyte multilayer coatings on titanium. Biomaterials.

[14] Huri PY, Huri G, Yasar U, Ucar Y, Dikmen N, Hasirci N, Hasirci V (2013). A biomimetic growth factor delivery strategy for enhanced regeneration of iliac crest defects. Biomed Mater.

[15] Wang H, Zou Q, Boerman OC, Nijhuis AW, Jansen JA, Li Y, Leeuwenburgh SC (2013). Combined delivery of BMP-2 and bFGF from nanostructured colloidal gelatin gels and its effect on bone regeneration in vivo. J Control Release.

[16] Hokugo A, Saito T, Li A, Sato K, Tabata Y, Jarrahy R (2014). Stimulation of bone regeneration following the controlled release of water-insoluble oxysterol from biodegradable hydrogel. Biomaterials.

[17] Kha HT, Basseri B, Shouhed D, Richardson J, Tetradis S, Hahn TJ, Parhami F (2004). Oxysterols regulate differentiation of mesenchymal stem cells: pro-bone and anti-fat. J Bone Miner Res.

[18] Johnson JS, Meliton V, Kim WK, Lee KB, Wang JC, Nguyen K, Yoo D, Jung ME, Atti E, Tetradis S, Pereira RC, Magyar C, Nargizyan T, Hahn TJ, Farouz F, Thies S, Parhami F (2011). Novel Oxysterols Have Pro-Osteogenic and Anti-Adipogenic Effects In Vitro and Induce Spinal Fusion In Vivo. J Cell Biochem.

[19] Chen C, Zhang SM, Lee IS (2013). Immobilizing bioactive molecules onto titanium implants to improve osseointegration. Surf Coat Tech.

[20] Son KM, Park HC, Kim NR, Lee IS, Yang HC (2010). Enhancement of the ALP activity of C3H10T1/2 cells by the combination of an oxysterol and apatite. Biomed Mater.

[21] Chen C, Qiu ZY, Zhang SM, Lee IS (2011). Biomimetic fibronectin/mineral and osteogenic growth peptide/mineral composites synthesized on calcium phosphate thin films. Chem Commun (Camb).

[22] Desai ES, Tang MY, Ross AE, Gemeinhart RA (2012). Critical factors affecting cell encapsulation in superporous hydrogels. Biomed Mater.

[23] D'Alessandro D, Pertici G, Moscato S, Metelli MR, Danti S, Nesti C, Berrettini S, Petrini M, Danti S (2014). Processing large-diameter poly(L-lactic acid) microfiber mesh/mesenchymal stromal cell constructs via resin embedding: an efficient histologic method. Biomed Mater.

[24] Ares MP, Porn-Ares MI, Thyberg J, Juntti-Berggren L, Berggren PO, Diczfalusy U, Kallin B, Bjorkhem I, Orrenius S, Nilsson J (1997). Ca2+ channel blockers verapamil and nifedipine inhibit apoptosis induced by 25-hydroxycholesterol in human aortic smooth muscle cells. J Lipid Res.

[25] Dugas B, Charbonnier S, Baarine M, Ragot K, Delmas D, Menetrier F, Lherminier J, Malvitte L, Khalfaoui T, Bron A (2010). Effects of oxysterols on cell viability, inflammatory cytokines, VEGF, and reactive oxygen species production on human retinal cells: cytoprotective effects and prevention of VEGF secretion by resveratrol. Eur J Nutr.

[26] Hokugo A, Sorice S, Parhami F, Yalom A, Li A, Zuk P, Jarrahy R (2013). A novel oxysterol promotes bone regeneration in rabbit cranial bone defects. J Tissue Eng Regen Med.

[27] Helmschrodt C, Becker S, Thiery J, Ceglarek U (2014). Preanalytical standardization for reactive oxygen species derived oxysterol analysis in human plasma by liquid chromatography-tandem mass spectrometry. Biochem Biophys Res Commun.

[28] Yang JX (2011). Study on biological functions of magnesium alloy/cobalt-chromium alloy surface coating. PhD thesis.

